# The rise of unmanned gyms: innovation, spatiotemporal characteristics, and the future of urban fitness

**DOI:** 10.3389/fspor.2025.1574966

**Published:** 2025-07-03

**Authors:** Tianhang Peng, Wanyuan Liang, Jiayi Zhang, Zike Zhang

**Affiliations:** ^1^ School of Sports Science, Beijing Sport University, Beijing, China; ^2^ School of Physical Education, University of Science and Technology Beijing, Beijing, China; ^3^ Key Laboratory of Physical Fitness and Exercise Rehabilitation of Hunan Province, Hunan Normal University, Changsha, China

**Keywords:** fitness industry, unattended fitness system, urban space, exercise time, sports industry

## Abstract

With the acceleration of urbanization and the growing awareness of health, traditional gyms face challenges related to space, time, and operational costs, particularly in meeting the fragmented fitness needs of urban residents. Unmanned gyms, integrating advanced technologies such as the Internet of Things (IoT) and Artificial Intelligence (AI), provide flexible, personalized fitness solutions available 24/7, addressing the increasing fitness demands of modern urban populations. Moreover, through innovative business models such as dynamic pricing, data monetization, and value-added services, unmanned gyms optimize their revenue structures and enhance user experiences. However, despite their significant growth potential, unmanned gyms still face challenges in terms of technology, ethics, and regulation. This paper reviews the current development of unmanned gyms, their spatiotemporal characteristics, and future trends. Future research should focus on the sustainability of technologies, user engagement, and the improvement of regulatory frameworks to foster the healthy growth of the unmanned gym industry.

## 1 Introduction

With the rapid pace of urbanization and the increasing awareness of health, initiatives such as “Healthy China” and “National Fitness” have emphasized the importance of fitness. The World Health Organization (WHO) highlighted in its “Global Action Plan on Physical Activity 2018–2030” that regular physical exercise can prevent non-communicable diseases (NCDs), hypertension, obesity, and improve mental health and quality of life (1). As fitness demand increases, particularly with the rise of unmanned gyms, the opportunities in the fitness industry have surged. Relevant policies indicate that improving the accessibility of fitness facilities is key to advancing national fitness plans (2). As urban populations continue to grow, it has become increasingly urgent to meet the diverse health, time, and location demands of individuals. Unmanned gyms, utilizing emerging technologies such as Internet of Things (IoT) and Artificial Intelligence (AI), provide flexible and cost-effective fitness solutions, addressing the fitness needs of urban residents. By overcoming the time and spatial constraints faced by traditional gyms, unmanned gyms offer innovative 24/7 fitness opportunities to busy urban populations (3). This study investigates the role of unmanned gyms as emerging components of urban health infrastructure. It aims to evaluate their technological integration, spatial and temporal accessibility, and operational models, and to assess their potential impact on urban health equity. Through a multidimensional analysis, this paper seeks to identify both the benefits and challenges of unmanned gym adoption, providing theoretical insights and practical implications for the design of sustainable urban fitness ecosystems.

## 2 Research methods

This study systematically examines the role of unmanned gyms in urban health infrastructure through a comprehensive analysis of existing literature, focusing on multiple dimensions including technological integration.

The literature search was conducted across several authoritative databases, including PubMed, Web of Science, Scopus, Google Scholar, and China National Knowledge Infrastructure (CNKI), covering publications from the inception of each database to the present. Relevant keywords such as “unmanned gyms,” “smart fitness,” “Internet of Things,” “artificial intelligence,” and “urban spatial accessibility” were employed using Boolean operators to refine the search. Additionally, citation tracking was utilized to ensure a thorough and systematic coverage of the literature. Inclusion criteria prioritized empirical studies, case analyses, and high-quality reviews focusing on technological applications, operational models, and social implications of unmanned gyms, published in reputable journals indexed by SCI, SSCI, or CSSCI. Studies exclusively addressing isolated technical components or non-peer-reviewed literature were excluded to maintain the scientific rigor and reliability of the findings. Full-text screening and relevant data extraction were performed on the selected articles. Thematic analysis was applied to synthesize key findings into three overarching themes: technological innovation, spatiotemporal characteristics, and socio-political impacts. This multidimensional analytical framework provides a robust theoretical foundation and practical guidance for advancing the understanding of unmanned gyms and their future development within urban fitness ecosystems.

## 3 Current development of unmanned gyms

### 3.1 Concept and characteristics

Building on previous research on unmanned gyms, this concept represents an innovative health service model driven by intelligent and automated technologies. It can be defined as a smart fitness space that integrates IoT technology, smart devices, environmental sensing, data interaction, and user self-service systems, offering fully automated management and personalized health solutions with the aim of reducing operational costs and enhancing user experience (4, 5). In this study, unmanned gyms are further defined as a new fitness service model deeply integrating emerging technologies such as the Internet of Things (IoT), Artificial Intelligence (AI), big data analytics, and computer vision.

The core characteristics of unmanned gyms are manifested in the use of intelligent hardware systems as the physical infrastructure (including smart access control, wearable devices, adaptive fitness equipment, etc.), along with real-time data interaction via environmental sensing modules (dynamic adjustment of temperature, humidity, and air quality) and user behavior capture systems (such as motion recognition, heart rate monitoring, and posture analysis), creating a “perception—analysis—response” feedback loop. Without the intervention of on-site staff, the entire process of user authentication [e.g., facial recognition or Quick Response(QR) code], workout plan customization (AI coaches), equipment parameter auto-adjustment (resistance/speed adjustments), energy consumption dynamic management (device sleep/wake modes), and emergency response (safety alerts) is automated through cloud-based algorithm platforms.

Typical application scenarios include 24/7 unmanned services, flexible payment models based on session or time usage, and continuous optimization of personalized health profiles based on users' biometric features and exercise data, forming a new fitness ecosystem that combines “smart equipment + digital services + precision operations.”

### 3.2 Global development trends

The global fitness industry is experiencing rapid growth, particularly in the areas of technological innovation and personalized health management, with future market trends indicating an increasing demand for tailored, refined services (6). Despite economic pressures, the global fitness industry continues to show strong growth prospects (7). The book “Digital Transformation of the Fitness Industry” points out that in the context of countries around the world jointly addressing challenges, the digitalization process of the fitness industry not only brings new challenges but also nurtures more opportunities (8). Unmanned gyms emerged in this context, initially in developed regions such as the United States and Europe, and are now expanding internationally. As public awareness of health and fitness services increases, unmanned gyms have seen widespread adoption. Notably, technologies such as smart fitness mirrors and AI-integrated mobile apps can provide real-time guidance and optimize user services.

In emerging markets like China, unmanned gyms have extended their reach into smaller cities and communities, accelerating the digital transformation and expansion of personalized services. Government support through national fitness policies has further accelerated the development of unmanned gyms (9).

### 3.3 Integration of core functions and key technological applications in unmanned gyms

As a major innovation in the fitness industry, unmanned gyms integrate intelligent technologies, automation, and personalized health services, aiming to address several core issues present in the operation of traditional gyms (5) (Table 1). The key features of unmanned gyms include: users can complete the entire process of booking, payment, and equipment control via a mobile app or smart devices without the need for human intervention. Smart access control systems enable entry via facial recognition or QR code scanning, while fitness equipment automatically powers on or off. All gym equipment is interconnected through IoT technology, allowing real-time upload of exercise data, which users can access and analyze anytime. Some devices can also automatically adjust resistance or speed based on user data, providing a more personalized training experience.

**Table 1:** The operational model differences between unmanned gyms and traditional gyms.

Dimensions	Traditional Gyms	Unmanned Gyms
Human Resource Dependency	Require front desk staff, trainers, cleaners, etc.	Fully automated; only require remote technical maintenance
Service Hours	Fixed operating hours (usually 8:00–22:00)	Open 24/7, flexible user scheduling
User Interaction	Rely on human instruction and class reservations	All reservations, payments, and data tracking done via APP
Cost Structure	High human resource costs, venue rent	Human resource costs reduced, smaller venue size
Degree of Personalization	Rely on trainer experience, mainly standardized classes	Precise personalization based on big data and AI
Safety and Hygiene	Rely on manual inspections	Sensors monitor equipment safety in real-time, ultraviolet disinfection is automated

Additionally, AI algorithms generate customized fitness plans based on user body data [such as Body Mass Index (BMI) and exercise habits], and real-time feedback and corrections are provided through an intelligent coaching system. IoT technology also enables real-time monitoring of equipment status, reducing operational and maintenance costs, while smart lighting and air conditioning systems adjust energy consumption based on foot traffic, thereby improving resource utilization. Unmanned gyms break the time limitations of traditional gyms, supporting 24/7 operations to meet the fragmented fitness needs of modern urban populations.

The development of smart portable devices opens up opportunities to conduct research, monitor health, and track disease with the vast amount of physiological, behavioral, and activity data that is constantly being collected in a variety of natural environments (10). From a technological application perspective, unmanned gyms fully leverage advanced technologies such as IoT, AI, and big data analytics. IoT technology enables fitness devices to synchronize user data to the cloud in real-time, while smart air conditioning and lighting systems automatically adjust based on foot traffic to reduce energy consumption. AI utilizes motion recognition technology to analyze users' exercise postures in real-time, providing corrective feedback. The virtual coach feature allows users to receive remote video guidance from coaches through the app, offering personalized training suggestions. Big data analytics integrates users' exercise data and health indicators, helping to generate long-term fitness trend reports and predict peak hours through historical data, dynamically adjusting maintenance cycles for equipment. The application of these technologies not only enhances the personalization and intelligence of fitness services but also significantly improves resource utilization efficiency and optimizes operational costs.

A systematic review of fitness center service quality reveals that traditional gyms are often affected by factors such as facility quality, staff quality, pricing, and accessibility, all of which can be improved to increase customer satisfaction (11). Compared to traditional gyms, the service model of unmanned gyms better mitigates the impact of these factors, effectively addressing issues such as high operational costs, fixed business hours, and limited revenue, providing users with greater flexibility. This is particularly suited to the fast-paced urban lifestyle, supporting the night-time economy and contributing to the development of low-carbon cities (12, 13). Intelligent technologies like facial recognition, AI coaching, and big data analytics make the training process more personalized by real-time adjustment of exercise intensity and offering 24/7 guidance, enhancing both convenience and effectiveness. The service quality of traditional gyms is often influenced by factors such as facility quality, staff competence, pricing, and accessibility, whereas unmanned gyms reduce the interference of these factors through technological innovations, offering a more efficient and cost-effective service model (14). Therefore, unmanned gyms not only possess advantages in technological application but also address many systemic issues of traditional gyms, holding significant market potential. These innovations, which integrate technology, urban planning, public health, and behavioral science, promote the development of smart cities, enhancing urban functionality and user satisfaction (15, 16).

## 4 Spatial accessibility

### 4.1 Health-oriented urban spatial planning and fitness accessibility

With urbanization accelerating, spatial planning is shifting from a “functional zoning” to a “health-oriented” approach. Optimizing urban space significantly impacts physical activity and public health, with community environments and infrastructure playing a crucial role in preventing chronic diseases (17, 18). In high-density cities, where space is limited and health demands are rising (19), improving the built environment can increase physical activity, particularly in areas with high accessibility to walking, cycling, and public transport facilities (20). In a study on health-oriented urban spatial planning and fitness accessibility, the dual value of running as a new commuting mode in promoting healthy lifestyles and urban livability is revealed, aiming to enhance the city's health accessibility and fitness convenience (21). Unmanned gyms optimize space utilization through smart technology, overcoming time and space constraints, reducing overcrowding during off-peak hours, and enhancing accessibility via mobile applications and smart devices (22). Their integration into transitional urban spaces—such as community centers and subway hubs—expands facility coverage within a 10-minute walk, fostering a more inclusive fitness culture and encouraging active lifestyles (23). Additionally, unmanned gyms provide a practical solution to increasing urban density, using compact and efficiently designed spaces to promote regular exercise without requiring large dedicated fitness areas.

### 4.2 Optimizing accessibility and urban fitness participation rates

Accessibility remains a critical determinant of fitness participation. Studies indicate that factors such as proximity to fitness facilities, transportation accessibility, and operating hours directly impact individuals' willingness to engage in exercise (24). Unmanned gyms strategically located in mid-to-high-rise residential areas optimize service efficiency, reducing both time and financial barriers to fitness, particularly for busy professionals and low-income populations (25). However, disparities persist, as high-quality fitness resources are often concentrated in affluent communities, whereas proximity to workplaces and commercial zones can mitigate sedentary behavior and increase participation in physical activity (26). By offering 24/7 access at lower operational costs, unmanned gyms bridge gaps in health resource distribution and contribute to social inclusion. Studies highlight that their flexible operating hours facilitate community interaction, improve social cohesion, and create an inclusive environment that encourages diverse participation (27). Moreover, integrating these gyms into mixed-use developments and commercial districts can attract diverse user groups, strengthening urban fitness networks and reinforcing a culture of health-conscious urban living.

## 5 Time flexibility

### 5.1 Benefits of morning exercise

Morning workouts are appealing to many urban working people because of its ability to start the day in a positive way. Early in the morning, when most people are still sleeping, a workout such as a gym session not only brings a sense of accomplishment, but also sets a positive tone for the entire day. Studies have found that the endorphins secreted in the body after exercise and the satisfaction of completing a task before 9 a.m. can greatly boost self-pleasure (28). Additionally, mornings can often be the only time of day that is not occupied by work, social or family activities, so choosing to work out in the morning can help avoid the “hard to find free time” of the day. According to Dr. Jagim of the Mayo Clinic, the sense of freedom and flexibility that comes with a morning workout not only reduces stress, but also helps to improve overall health (29). After a night of energy consumption, the body's glycogen reserves have been depleted, this time to exercise can be greater use of fat for energy supply, may be more helpful for fat loss.

### 5.2 Advantages of night-time fitness and health outcome analysis

The fast-paced lifestyle of modern urban workers, characterized by long hours of high-pressure work, significantly reduces physical activity levels (30), leading to health issues such as obesity (31). Evidence-based medical research indicates that periodic and systematic exercise can improve the sleep-wake cycle and metabolic homeostasis by regulating the expression of biological clock genes (32). Research based on Information Structure Modeling (ISM) and Complex Data Analysis (CoDA) suggests that physical activity effectively reduces psychological stress and enhances mental health (33). Exercise interventions are considered an effective non-pharmacological method for improving sleep quality and mental health in patients with chronic insomnia (34). Exercise at different times of day has varying effects on weight control and metabolism (35), and evening aerobic exercise significantly improves blood pressure and heart rate recovery in hypertensive patients (36). While some studies suggest that the time of exercise has little impact on sleep quality (37, 38), other studies highlight that exercise can help adjust sleep structure and improve immune and nervous system function (39). Therefore, night-time exercise may have positive effects on health. Unmanned gyms, by offering flexible time options, make night-time fitness possible, meeting the fitness needs of night-shift workers, and becoming an important part of the urban night-time economy (40).

## 6 Future development

### 6.1 Operational model optimization and data-driven decision making

Unmanned gyms can be optimized through data-driven decision-making systems that integrate urban planning, environmental features, and user behavior data. Intelligent management systems enhance resource utilization and optimize spatial layout to improve accessibility and space efficiency. For instance, pedestrian heat maps can be used to reduce congestion within gyms, enhancing the overall user experience (41, 42). Awais (2024) demonstrated that AI-driven dynamic pricing models can adjust membership fees based on real-time demand, improving off-peak usage rates (43). These models not only balance operational workload but also provide more cost-effective choices for users, boosting satisfaction. Moreover, the integration of smart mirrors and wearable devices allows users to receive real-time feedback, further customizing their workout plans. A case study showed that users utilizing AI feedback systems had significantly higher workout frequencies compared to traditional gym users (44).

### 6.2 Revenue model innovation and consumer behavior analysis

Revenue model innovation in unmanned gyms is seen in three key areas. First, dynamic membership pricing uses reinforcement learning models to adjust fees based on user demand and equipment usage, effectively increasing off-peak usage rates (45). Second, innovation in data monetization pathways involves partnerships with health insurance companies, linking real-time user activity data to premium discounts. This not only generates additional revenue for gyms but also strengthens user engagement (46). Third, value-added services like 3D posture scans and AI nutrition plans cater to users' demands for personalized fitness and health management, while providing significant non-membership revenue. This diversified revenue structure offers new commercialization paths for the fitness industry and enhances the user experience (46).

Consumer motivations for adopting unmanned gyms are multi-layered, with time flexibility and privacy needs being core drivers, especially among younger demographics. Most users view “avoiding peak hours” and “no human trainer intervention” as key decision-making factors, which align closely with the autonomy needs outlined in self-determination theory (SDT) (47). Gender differences are apparent, with more than half of female users preferring computer-vision-based posture correction services, while male users tend to favor strength training metrics like barbell speed tracking. Despite these preferences, the Technology Acceptance Model (TAM) shows that some potential users are skeptical about AI replacing human trainers, particularly in preventing injuries during complex movements (such as CrossFit high-intensity training), highlighting the importance of perceived usefulness and ease of use in user decisions (48, 49).

### 6.3 Technological breakthroughs and ethical considerations

Unmanned gyms have achieved three key technological breakthroughs: First, edge computing technology processes most data locally, reducing reliance on cloud computing and bandwidth costs, crucial for real-time tracking and emergency alerts. Second, decentralized data storage technologies significantly lower the risk of privacy breaches by minimizing data sharing across gyms (50, 51). Third, the introduction of smart contracts allows for the automated execution of membership agreements, reducing operational disputes and costs (46). However, ethical concerns remain, especially regarding inclusivity and transparency. Research shows that current exercise algorithms can only accurately identify a limited number of wheelchair users, exposing a lack of inclusivity in design (52). Furthermore, many users are unaware of how their biometric data is monetized, raising concerns over data usage transparency. Moving forward, stronger data privacy protections and inclusive design must be prioritized to ensure that all users can equally benefit from the technological innovations of unmanned gyms.

### 6.4 Regulatory framework and policy implications

Currently, there are significant regional differences in the regulatory frameworks governing unmanned gyms. The European Union's Artificial Intelligence Liability Directive mandates that high-risk AI systems must include error traceability modules; for example, motion recognition algorithms are required to maintain decision logs (53). In contrast, China's Guidelines for Unmanned Business Management primarily focus on physical safety standards, while data governance provisions still need further refinement (54). To foster the healthy development of unmanned gyms, a dual-track regulatory system is recommended. First, the implementation of ISO 42001-certified AI audit protocols should be enforced to ensure algorithm transparency and monitor bias rates. Second, referring to Japan's assistive device subsidy policies, VAT exemptions should be granted to companies engaged in the development of accessible equipment, a practice that has already proven effective in increasing equipment adoption rates (55).

### 6.5 Challenges and future research directions

Despite the vast potential of unmanned gyms, three core challenges remain. First, the long-term feasibility of artificial intelligence and Internet of Things (IoT) systems requires substantial research and development investment. Second, real-time injury prevention mechanisms, particularly in complex exercises such as CrossFit, need further refinement. Lastly, enhancing user retention and maintaining engagement through gamification and social features is crucial for the long-term success of unmanned gyms. Future research should employ a mixed-methods approach to strengthen empirical analysis. Longitudinal studies could track users' long-term behavior, while ethnographic analysis could identify cultural barriers to gamification adoption in different regions. Moreover, policy simulation studies can evaluate the impact of regulatory changes on market dynamics, providing valuable data support for government and industry decision-makers.

## 7 Conclusion

**Figure 1:**
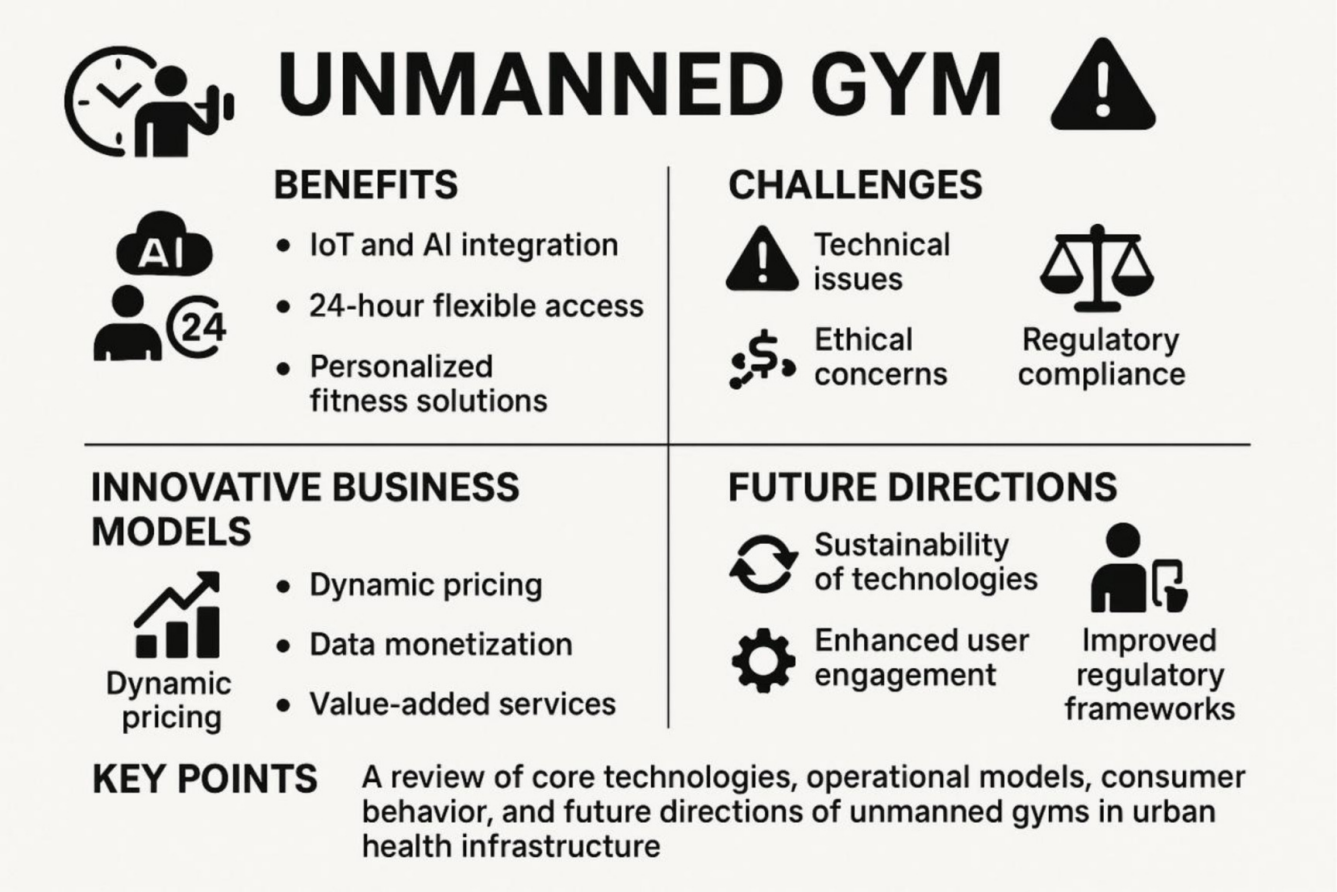
Overview of unmanned gyms: this diagram integrates the internet of things (IoT) and artificial intelligence (AI) technologies to provide flexible and personalized fitness solutions available 24/7. The figure highlights the main advantages, innovative business models, challenges, and future development directions of unmanned gyms.

As an innovative service model within the fitness industry, unmanned gyms have demonstrated significant market potential and play a crucial role in addressing the diverse fitness needs of urban residents, optimizing operational costs, and enhancing user experience (Figure 1). Through intelligent equipment and personalized training systems, unmanned gyms not only alleviate the time and space limitations faced by traditional gyms but also break the constraints of high costs and fixed schedules. However, despite the unprecedented advantages offered by technological innovations, ethical issues such as data privacy and inclusive design still require further attention. Furthermore, the current regulatory frameworks are not yet harmonized globally, necessitating cross-national cooperation among policymakers to establish unified regulatory standards that will ensure the sustainable development of the industry. Future research should focus on the long-term impact of unmanned gyms on urban health, explore ways to overcome existing technological and social barriers, and develop more effective mechanisms for user retention and social inclusivity.This study integrates literature on the technical architecture of the fitness industry and user behavior analysis, offering the first systematic exploration of unmanned gyms, revealing their social benefits and potential challenges. Although it fills a research gap, it is limited by insufficient literature in the emerging field, with current conclusions mainly relying on cross-sectional data and lacking longitudinal tracking and multi-center cohort studies. Future research should focus on long-term tracking to uncover the mechanisms of user retention, and interdisciplinary collaboration should be pursued to analyze the interactions between technology and social factors, providing a solid scientific basis and policy support for the sustainable development of urban fitness ecosystems.

## References

[1] World Health Organization. Global Action Plan on Physical Activity 2018–2030: More Active People for a Healthier World (2018). Available online at: https://www.who.int/publications/i/item/9789241514187 (Accessed February 5, 2025).

[2] State Council of China. Circular of the State Council on the Issuance of the National Fitness Program (2021–2025) (Gazette of the State Council No. 23). (2021). Available online at: https://www.gov.cn/gongbao/content/2021/content_5631816.htm (Accessed May 26, 2025).

[3] Zhang H. A comparative study of business strategies between 24-hour gyms and traditional gyms based on the SWOT model: take LeFit as an example. Adv Econ Manage Politic Sci. (2023) 37(1):219–26. 10.54254/2754-1169/37/20231864

[4] Ye S Song Q. Research on the business model of internet-based unattended smart gym. Innov Entrep. (2022) 6(2):71–2.

[5] Lin D. Digital transformation of gyms in the context of digital economy. China Market. (2023) 15:193–6.

[6] Chekhovska L. Fitness industry: state and prospects of development in the countries of the world. Slobozhanskyi Science and Sport Bulletin. (2017) 58(2):107–12. Available online at: https://journals.uran.ua/sport_herald/article/view/104259

[7] Thompson WR. Worldwide Survey of Fitness Trends for 2023 (2023).

[8] Pedragosa V Barbosa HF. Digital transformation and innovation in Portugal fitness centres. In: García-Fernández J Valcarce-Torrente M Mohammadi S, editors. The Digital Transformation of the Fitness Sector: A Global Perspective. Bingley: Emerald Publishing (2022). p. 23–9.

[9] Zhu KY. Research on the development of intelligent gyms under the national fitness strategy. China Sports Science Society Conference Proceedings (2018). p. 482–3

[10] Shandhi MM Singh K Janson N Ashar P Singh G Lu B Assessment of ownership of smart devices and the acceptability of digital health data sharing. NPJ Digital Med. (2024) 7(1):44. 10.1038/s41746-024-01030-x PMC10883993 38388660

[11] Barbosa HF Barbosa J Sabino B Loureiro V. Determinants of service quality influencing customer satisfaction in fitness centers: a systematic review.European journal of human movement. Eur J Hum Mov. (2022) 49:29–45. 10.21134/eurjhm.2022.49.3

[12] Li Z. Research on the Business Model of Intelligent Gym in the Era of Internet+ (D). Beijing: Beijing Sport University (2019).

[13] Li XG. Investigation and countermeasures research on member satisfaction of 24-hour self-service gyms in Nanjing: a case study of Lekefitness in Pukou district. Contemp Sports Technol. (2021) 11(30):124–31. 10.16655/j.cnki.2095-2813.2103-1579-2326

[14] Bhatia M Sood SK. An intelligent framework for workouts in gymnasium: m-health perspective. Comput ElectrEng. (2018) 65:292–309. 10.1016/j.compeleceng.2017.07.018

[15] Kohl HW Craig CL Lambert EV Inoue S Alkandari JR Leetongin G The pandemic of physical inactivity: global action for public health. Lancet. (2012) 380(9838):294–305. 10.1016/S0140-6736(12)60898-8 22818941

[16] Gjestvang C Tangen EM Arntzen MB Haakstad LA. How do fitness club members differentiate in background characteristics, exercise motivation, and social support? A cross-sectional study. J Sports Sci Med. (2023) 22(2):235–44. 10.52082/jssm.2023.234 37293418 PMC10244985

[17] Douglas M. Review of’ shaping neighbourhoods for local health and global sustainability'. J Public Health. (2010) 32(4):589. 10.1093/pubmed/fdq077

[18] Congdon P. Obesity and urban environments. Int J Environ Res Public Health. (2019) 16(3):464. 10.3390/ijerph16030464 30764541 PMC6388392

[19] Sallis JF Cerin E Conway TL Adams MA Frank LD Pratt M Physical activity in relation to urban environments in 14 cities worldwide: a cross-sectional study. Lancet. (2016) 387(10034):2207–17. 10.1016/s0140-6736(15)01284-2 27045735 PMC10833440

[20] Kärmeniemi M Lankila T Ikäheimo T Koivumaa-Honkanen H Korpelainen R. The built environment as a determinant of physical activity: a systematic review of longitudinal studies and natural experiments. Ann Behav Med. (2018) 52(3):239–51. 10.1093/abm/kax043 29538664

[21] Anagnostopoulos A. The rise of run-commuting as a form of transportation: research on the characteristics and spatial needs of these trips. In: Nathanail E.G. Adamos G. Karakikes I., editors, Advances in Mobility-as-a-Service Systems. CSUM 2020. Advances in Intelligent Systems and Computing, vol 1278; Cham: Springer (2021).

[22] Running at Night in the City: The Phenomenon of “Night Runners” in Coimbra.

[23] Nieuwenhuijsen MJ. New urban models for more sustainable, liveable and healthier cities post covid19; reducing air pollution, noise and heat island effects and increasing green space and physical activity. Environ Int. (2021) 157:106850. 10.1016/j.envint.2021.106850 34531034 PMC8457623

[24] Sallis JF Saelens BE Frank LD Conway TL Slymen DJ Cain KL Neighborhood built environment and income: examining multiple health outcomes. Soc Sci Med. (2009) 68(7):1285–93. 10.1016/j.socscimed.2009.01.017 19232809 PMC3500640

[25] Mohd Aznan EA Ismail AD Mohd Kassim AF Md Yusof MK Samat HA. Accessibility of fitness facilities and physical activity participation: a comparison of disabled athletes and disabled people. J Intelek. (2022) 17(1):211. 10.24191/ji.v17i1.16040

[26] Lin C-Y Koohsari MJ Liao Y Ishii K Shibata A Nakaya T Workplace neighbourhood built environment and workers’ physically-active and sedentary behaviour: a systematic review of observational studies. Int J Behav Nutr Phys Act. (2020) 17(1):148. 10.1186/s12966-020-01055-x 33218343 PMC7678125

[27] Grigoletto A Mauro M Maietta Latessa P Iannuzzi V Gori D Campa F Impact of different types of physical activity in green urban space on adult health and behaviors: a systematic review. Eur J Investig Health Psychol Educ. (2021) 11(1):263–75. 10.3390/ejihpe11010020 34542463 PMC8314339

[28] Feng H Yang L Liang YY Ai S Liu Y Liu Y Associations of timing of physical activity with all-cause and cause-specific mortality in a prospective cohort study. Nat Commun. (2023) 14(1):930. 10.1038/s41467-023-36546-5 36805455 PMC9938683

[29] Exercise Timing Conundrum: Optimal Workout Timing. Rick Thiesse. Available online at: https://newsnetwork.mayoclinic.org/discussion/exercise-timing-conundrum-optimal-workout-timing/

[30] Müller AM Chen B Wang NX Whitton C Direito A Petrunoff N Correlates of sedentary behaviour in Asian adults: a systematic review. Obes Rev. (2020) 21(4):e12976. 10.1111/obr.12976 31919972

[31] Kracht CL Burkart S Groves CI Balbim GM Pfledderer CD Porter CD 24-hour Movement behavior adherence and associations with health outcomes: an umbrella review. J Act Sedent Sleep Behav. (2024) 3(1):25. 10.1186/s44167-024-00064-6 PMC11467106 39399355

[32] Ceylan Hİ Saygın Ö Özel Türkcü Ü. Assessment of acute aerobic exercise in the morning versus evening on asprosin, spexin, lipocalin-2, and insulin level in overweight/obese versus normal weight adult men. Chronobiol Int. (2020) 37(8):1252–68. 10.1080/07420528.2020.1792482 32741294

[33] Chekroud SR Gueorguieva R Zheutlin AB Paulus M Krumholz HM Krystal JH Association between physical exercise and mental health in 1·2 million individuals in the USA between 2011 and 2015: a cross-sectional study. Lancet Psychiatry. (2018) 5(9):739–46. 10.1016/S2215-0366(18)30227-X 30099000

[34] Abd El-Kader SM Al-Jiffri OH. Aerobic exercise affects sleep, psychological wellbeing and immune system parameters among subjects with chronic primary insomnia. Afr Health Sci. (2020) 20(4):1761–9. 10.4314/ahs.v20i4.29 34394237 PMC8351861

[35] Creasy SA Wayland L Panter SL Purcell SA Rosenberg R Willis EA Effect of morning and evening exercise on energy balance: a pilot study. Nutrients. (2022) 14(4):816. 10.3390/nu14040816 35215466 PMC8877501

[36] Brito LC Peçanha T Fecchio RY Pio-Abreu A Silva G Mion-Junior D Comparison of morning versus evening aerobic-exercise training on heart rate recovery in treated hypertensive men: a randomized controlled trial. Blood Press Monit. (2021) 26(5):388–92. 10.1097/MBP.0000000000000545 34001759

[37] Burgess VN Antonio J Bland HW Wagner R Tartar JL Melton BF. The effect of timing and type of exercise on the quality of sleep in trained individuals.international. J Exerc Sci. (2020) 13(7):837–58. 10.70252/BKKE7434 PMC7449340 32922649

[38] Miller DJ Sargent C Roach GD Scanlan AT Vincent GE Lastella M. Moderate-intensity exercise performed in the evening does not impair sleep in healthy males. Eur J Sport Sci. (2020) 20(1):80–9. 10.1080/17461391.2019.1611934 31072217

[39] Gong MJ Tang Q Tan SJ Hu X. Research progress on the effects and mechanisms of exercise intervention on sleep disorders. J Sichuan Univ. (2024) 55(1):236–42. 10.12182/20240160404 PMC10839477 38322540

[40] Du K Zhang YA. The temporal and spatial dimensions of urban white-collar nighttime fitness from the perspective of a singular society: a field study based on 24-hour unmanned gyms. Proceedings of the 12th National Sports Science Conference; Rizhao, Shandong, China (2022). p. 130–1

[41] Abbott AA. Facility layout and maintenance concerns. ACSM’s Health Fit J. (2018) 22(1):37. 10.1249/FIT.0000000000000356

[42] Wan Y Tang H. Design of optimization algorithm for configuration of amateur sports training equipment in smart city community. Comput Intell Neurosci. (2022) 2022:9572395. 10.1155/2022/9572395 35785058 PMC9246622

[43] Lightsey HM Maier SP Bono CM Kang JD Harris MB. In-hospital, 24-hour exercise spaces for resident and staff wellness. HSS J. (2023) 19(2):140–5. 10.1177/15563316221131031 37065098 PMC10090845

[44] Park H Lee G Seo B Billinghurst M. User experience design for a smart-mirror-based personalized training system. Multimed Tools Appl. (2021) 80:31159–81. 10.1007/s11042-020-10148-5

[45] Awais M. Summer 2024. Qlantic J Soc Sci. (2024) 5(3):99–108. 10.55737/qjss.370771519

[46] Zhigang B. Research on the Development Strategy of Kindred Egg Sports Intelligent Gymnasium Under the Background of “Internet+”. Tianjin: Tianjin University (2020).

[47] Smidt N de Vet HC Bouter LM Dekker J Arendzen JH de Bie RA Effectiveness of exercise therapy: a best-evidence summary of systematic reviews. Aust J Physiother. (2005) 51(2):71–85. 10.1016/S0004-9514(05)70036-2 15924510

[48] Teixeira PJ Carraça EV Markland D Silva MN Ryan RM. Exercise, physical activity, and self-determination theory: a systematic review. Int J Behav Nutr Phys Act. (2012) 9:78. 10.1186/1479-5868-9-78 22726453 PMC3441783

[49] Wenjun P. Research on the influence of perceived value of exercise data on fitness course experience and course repurchase intention under technology acceptance model. J Zunyi Norm Coll. (2023) 25(1):152–5.

[50] Chunhua S. Analysis of marketing strategies of sports social platforms–taking keep as an example. E-Commerce Rev. (2024) 13(3):5278–93.

[51] Liu X Lin K. The protection of data privacy for smart wearable device users in the context of digitalization transformation of national fitness. J Phys Educ. (2024) 31(4):64–72.

[52] Huang P Xu L Xie Y. Biomedical applications of electromagnetic detection: a brief review. Biosensors. (2021) 11(7):225. 10.3390/bios11070225 34356696 PMC8301974

[53] European Commission. Proposal for a directive on artificial intelligence liability (AI liability directive). [COM(2023) 18 final]. Official J Eur Union. (2023). C 77:6–8. Available online at: https://www.europarl.europa.eu/RegData/etudes/BRIE/2023/739342/EPRS_BRI(2023)739342_EN.pdf

[54] State Administration for Market Regulation (SAMR). Guidelines for Unmanned Commercial Management in China (2021). Available online at: https://english.www.gov.cn/news/pressbriefings/202108/25/content_WS61264d18c6d0df57f98df269.html (Accessed August 25, 2021).

[55] Zhu K Li CQ. International experiences and enlightenment of financing for assistive devices for persons with disabilities. Chin J Rehabil Theory Pract. (2011) 17(11):1090–2.

